# Two hundred and five newly assembled mitogenomes provide mixed evidence for rivers as drivers of speciation for Amazonian primates

**DOI:** 10.1111/mec.16554

**Published:** 2022-06-20

**Authors:** Mareike C. Janiak, Felipe E. Silva, Robin M. D. Beck, Dorien de Vries, Lukas F. K. Kuderna, Nicole S. Torosin, Amanda D. Melin, Tomàs Marquès‐Bonet, Ian B. Goodhead, Mariluce Messias, Maria N. F. da Silva, Iracilda Sampaio, Izeni P. Farias, Rogerio Rossi, Fabiano R. de Melo, João Valsecchi, Tomas Hrbek, Jean P. Boubli

**Affiliations:** ^1^ School of Science, Engineering & Environment University of Salford Salford UK; ^2^ Research Group on Primate Biology and Conservation Mamirauá Institute for Sustainable Development Tefé AM Brazil; ^3^ Unit of Evolutionary Biology and Ecology (EBE), Département de Biologie des Organismes Université Libre de Bruxelles Brussels Belgium; ^4^ Institute of Evolutionary Biology (UPF‐CSIC) Barcelona USA; ^5^ Department of Genetics Human Genetics Institute of New Jersey Rutgers University Piscataway New Jersey USA; ^6^ Department of Anthropology & Archaeology and Department of Medical Genetics University of Calgary Calgary Alberta Canada; ^7^ Alberta Children's Hospital Research Institute Calgary Alberta Canada; ^8^ Catalan Institution of Research and Advanced Studies (ICREA) Barcelona Spain; ^9^ CNAG‐CRG, Centre for Genomic Regulation (CRG) Barcelona Institute of Science and Technology (BIST) Barcelona Spain; ^10^ Institut Català de Paleontologia Miquel Crusafont Universitat Autònoma de Barcelona, Edifici ICTA‐ICP Cerdanyola del Vallès, Barcelona Spain; ^11^ Department of Biology Universidade Federal de Rondônia Porto Velho RO Brazil; ^12^ Coleção de Mamíferos Instituto Nacional de Pesquisas da Amazônia Manaus AM Brazil; ^13^ Universidade Federal do Pará Belém PA Brazil; ^14^ Laboratory of Evolution and Animal Genetics Universidade Federal do Amazonas Manaus AM Brazil; ^15^ Instituto de Biociências Universidade Federal do Mato Grosso Cuiabá MT Brazil; ^16^ Department of Forestry Engineering Universidade Federal de Viçosa Viçosa MG Brazil; ^17^ Department of Biology Trinity University San Antonio Texas USA; ^18^ Present address: Illumina Artificial Intelligence Laboratory Illumina Inc. San Diego CA USA

**Keywords:** mitochondrial DNA, molecular phylogenetics, platyrrhines, riverine barrier hypothesis, South American primates

## Abstract

Mitochondrial DNA remains a cornerstone for molecular ecology, especially for study species from which high‐quality tissue samples cannot be easily obtained. Methods using mitochondrial markers are usually reliant on reference databases, but these are often incomplete. Furthermore, available mitochondrial genomes often lack crucial metadata, such as sampling location, limiting their utility for many analyses. Here, we assembled 205 new mitochondrial genomes for platyrrhine primates, most from the Amazon and with known sampling locations. We present a dated mitogenomic phylogeny based on these samples along with additional published platyrrhine mitogenomes, and use this to assess support for the long‐standing riverine barrier hypothesis (RBH), which proposes that river formation was a major driver of speciation in Amazonian primates. Along the Amazon, Negro, and Madeira rivers, we found mixed support for the RBH. While we identified divergences that coincide with a river barrier, only some occur synchronously and also overlap with the proposed dates of river formation. The most compelling evidence is for the Amazon river potentially driving speciation within bearded saki monkeys (*Chiropotes* spp.) and within the smallest extant platyrrhines, the marmosets and tamarins. However, we also found that even large rivers do not appear to be barriers for some primates, including howler monkeys (*Alouatta* spp.), uakaris (*Cacajao* spp.), sakis (*Pithecia* spp.), and robust capuchins (*Sapajus* spp.). Our results support a more nuanced, clade‐specific effect of riverine barriers and suggest that other evolutionary mechanisms, besides the RBH and allopatric speciation, may have played an important role in the diversification of platyrrhines.

## 1 INTRODUCTION

Although the number of whole genomes available for nonmodel organisms has grown dramatically, mitochondrial DNA (mtDNA) remains a cornerstone for many areas of research, including species diversification dynamics, phylogenetics, and conservation genetics (Cardeñosa et al., 2021; Flores‐Manzanero et al., 2022; Reese et al., 2020; Schmidt et al., 2018; Serrao et al., 2018), especially for study species from which high‐quality tissue samples cannot be easily obtained. Difficulties with invasive sampling for high‐quality tissues or blood include practical issues with trapping large‐bodied, arboreal, or marine animals, as well as ethical considerations, such as risks to the animal and to researchers (Aristizabal Duque et al., 2018). These difficulties apply to collecting invasive samples from most primates, and many genetic and genomic studies in primatology continue to rely on materials that can be collected noninvasively (Arandjelovic & Vigilant, 2018; Aylward et al., 2018; Orkin et al., 2016), or on historic samples, such as from museum skins (Burrell et al., 2015). Although there have been methodological advances that allow for the retrieval of nuclear DNA and even whole genomes from these materials (Burrell et al., 2015; Chiou & Bergey, 2018; Fontsere et al., 2021; Orkin et al., 2021), mtDNA continues to be the most accessible and cost‐effective source of genetic data.

Mitochondrial markers and genomes are especially useful for species identification and delimitation (Reese et al., 2020), for assessing population structure (Flores‐Manzanero et al., 2022; Gagneux et al., 1999; Phukuntsi et al., 2021; Serrao et al., 2018; Skovrind et al., 2021), for assessing introgression and admixture (Makhov et al., 2021; Malukiewicz et al., 2021), for monitoring of species assemblages using environmental DNA (Barnes & Turner, 2016; Thomsen & Willerslev, 2015), and for identifying the origin of animals found in wild meat markets and the illegal pet trade (Cardeñosa et al., 2021; Maligana et al., 2020; Russello et al., 2008). However, many of these methods are reliant on databases from which sequences can be integrated and against which results can be compared, and which are often incomplete (Curry et al., 2018). For example, for platyrrhine primates (a group including all monkeys found in Central and South America) only 32 mitochondrial genome assemblies are available in RefSeq, even though over 200 species have been described, and complete platyrrhine mitogenomes are only available for 76 individuals in GenBank overall. Additionally, the majority of these mitogenomes contain little or no metadata, such as sampling locality, limiting their utility for many analyses, including population genetic studies that rely on spatial data (Deichmann et al., 2017; Strohm et al., 2016; Tahsin et al., 2016).

Hypotheses about how landscape features have shaped the distribution and richness of species can be investigated with molecular data that include sampling localities, and mitochondrial DNA is a fast‐evolving marker (Brown et al., 1979), which can shed light on evolutionary relationships within young radiations more quickly than nuclear DNA. As such, mitogenomic data sets may be especially useful for assessing biogeographic and phylogeographic questions. Primates found within the Amazon are disproportionately speciose for the geographic area they occupy (Fordham et al., 2020), and Alfred Russel Wallace noted that the distributions of many Amazonian primates appear to be limited by boundaries formed by the Amazon, Madeira, and Negro rivers (Wallace, 1852). Now known as the riverine barrier hypothesis (RBH), it is a long‐standing paradigm used to explain the extraordinary species richness of not just primates (Ayres & Clutton‐Brock, 1992; Boubli et al., 2015), but also other mammals (Patton et al., 1994), birds (Cracraft, 1985; Hayes & Sewlal, 2004; Pomara et al., 2014), amphibians and reptiles (de Fraga & de Carvalho, 2021; Godinho & da Silva, 2018; Ortiz et al., 2018), and butterflies (Hall & Harvey, 2002). The RBH proposes that the rivers of the Amazon river basin acted as drivers of speciation when their formation divided existing species' ranges and formed barriers to continued gene flow, leading to allopatric speciation. As an extension of the RBH, the Amazon has been divided into proposed areas of endemism: interfluvial regions which are suggested to harbour unique species assemblages, and which have been used as units for conservation planning (da Silva et al., 2005). However, the RBH and proposed areas of endemism are not without controversy. Criticisms include limits of interspecific phylogenetic comparative methods, and that many studies are based on very few taxa or single gene markers (Losos & Glor, 2003; Santorelli et al., 2018). In addition, some large‐scale studies have found little or only species‐specific support for the RBH (Dambros et al., 2020; Gascon et al., 2000; Kopuchian et al., 2020; Naka & Brumfield, 2018; Santorelli et al., 2018; Smith et al., 2014).

**FIGURE 1:**
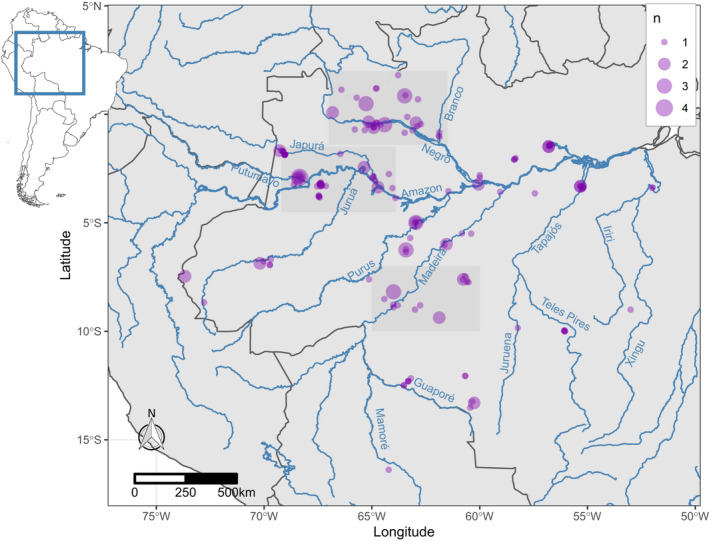
Sample locations of Amazonian primates included in this study. Point size reflects number of samples available from the same location. Major rivers that are relevant for this study are labelled. Shaded regions identify areas explored in detail in Figure 5

Here, we assemble more than 200 new mitochondrial genomes for Amazonian primates, with locality information (Figure 1), combine these with other Amazonian primate mitogenomes currently available, and use this data set to produce a dated phylogeny (“timetree”), which we use to assess support for the RBH. Specifically, we explore support for rivers as engines of speciation by first identifying divergences in the mitochondrial phylogeny where members of the neighbouring clades are found only on opposite sides of the major river boundaries proposed by Wallace (Amazon, Negro, and Madeira rivers; (1852)), followed by assessing synchrony of congruent divergences occurring for the same river and comparing these dates to current geological evidence for the timing of river formation. We consider divergences to be congruent with the RBH if divergences meet both conditions, namely that (1) sister taxa are found only on opposite sides of a river and that (2) the timing of the divergence does not postdate the geological estimate of river formation.

## 2 MATERIALS AND METHODS

### 2.1 Sample acquisition

We obtained wild‐caught primate tissue samples stored in the following Brazilian zoological collections: Instituto Nacional de Pesquisas da Amazônia (INPA), Universidade Federal do Amazonas (UFAM), Instituto de Desenvolvimento Sustentável Mamirauá (IDSM), Museu Nacional do Rio de Janeiro (MN), Museu Paraense Emílio Goeldi (MPEG), Universidade Federal de Rondônia (UFRO), Universidade Federal do Mato Grosso (UFMT) (Table S1). The majority of these samples were collected during multiple large field surveys aimed at surveying Amazonian biodiversity which were commissioned by the Brazilian government (e.g., PROBIO, SISBIOTA) from 2000 to 2017, while others were obtained from animals hunted by local communities as part of monitoring programmes. Samples consisted of muscle tissue preserved in 70%–90% ethanol. The acquisition of samples for *Alouatta caraya* and *Alouatta guariba clamitans* from Argentina has been previously described (Torosin, Argibay, et al., 2020; Torosin, Webster, et al., 2020). Samples from *Alouatta palliata*, *Ateles geoffroyi*, *Saimiri oerstedii*, and *Saguinus geoffroyi* were biobanked at Kids Saving the Rainforest (KSTR), a wildlife rehabilitation facility in Quepos, Costa Rica in 2016–2017. The *A*. *geoffroyi*, *A*. *palliata* and *S. oerstedii* individuals were wild‐born individuals that were brought to KSTR due to injuries or recovered from the pet trade. The *Saguinus geoffroyi* individual was surrendered to KSTR from a private collection of unknown origin (this species is not native to Costa Rica).

In Brazil, collection permits were obtained from the Biodiversity Authorization and Information System (SISBIO; permit nos. 55777, 42111, 32095–1, 7795–1) and exported under CITES permits (19BR033597/DF and15BR019039/DF). Costa Rican samples were collected and exported under permits from the Comisión Nacional para la Gestión de la Biodiversidad (R‐002‐2020‐OT‐CONAGEBIO) and CITES (2016‐CR2392/SJ [no. S 2477]; 2020‐CR‐4889/SJ [no. S 6825]).

### 2.2 Sample extraction, sequencing, and mitochondrial genome assembly

Sample extraction and sequencing for samples AC_t1 and AGC_m1 (see Table S1) were previously described (Torosin, Argibay, et al., 2020a; Torosin, Webster, et al., 2020b). Details on genomic sequence generation for the remaining samples are provided in Kuderna et al. (2022). Briefly, genomic DNA was extracted and libraries prepared using standard Illumina protocols and libraries were sequenced to ~30× coverage on an Illumina NovaSeq6000 (150 bp paired‐end reads). Reads were trimmed to remove any sequencing adapters or primers with cutadapt version 2.10 (Martin, 2011) and then subsampled to 3.5 million read pairs with reformat.sh from the bbtools suite v38.86 (Bushnell, 2014). We used mitofinder version 1.4 (Allio et al., 2020) to assemble and annotate mitochondrial genomes from the trimmed and subsampled Illumina short reads, using metaspades (Nurk et al., 2017) for the assembly step and mitfi (Jühling et al., 2012) for the tRNA annotation step. If multiple mitochondrial contigs were identified, we ran mitofinder a second time, setting the minimum contig size to 10,000 and the maximum contigs to 1, in order to force selection and annotation of only the single best contig. For each sample, we used the complete mitochondrial genome from a closely related species available in NCBI's RefSeq database as the reference genome in mitofinder (Supporting Information). All mitochondrial genomes were compared to the ncbi reference database via blast searches to confirm correct taxon identity and to check for completeness.

### 2.3 Alignment, trimming, and partitioning

We aligned mitochondrial genomes newly assembled with mitofinder (*n* = 205), as well as complete mitochondrial genomes from 32 additional platyrrhines and six primate outgroups (Table S1) available in ncbi's RefSeq database from previous phylogenomic studies (Arnason et al., 2000, 2002; Babb et al., 2011; Chan et al., 2010; Chiou et al., 2011; de Freitas et al., 2018; Finstermeier et al., 2013; Hao & Yi, 2019; Hodgson et al., 2009; Horai et al.,^1995^; Malukiewicz et al., 2017, 2021; Matsui et al., 2009; Menezes et al., 2013; Raaum et al., 2005; Wang et al., 2016; Zhang et al., 2016). To facilitate the alignment of circular genomes, we first shifted the genome start for all sequences to begin with the gene cytochrome B, using the fasta_shift tool (https://github.com/b‐brankovics/fasta_tools). The shifted sequences were aligned with mafft v7.309 (Katoh & Standley, 2013). We trimmed the resulting alignment with trimal v1.2 (Capella‐Gutiérrez et al., 2009) using the gappyout setting. Following (Hassanin et al., 2021), we retained only the 12 protein‐coding genes on the forward (“heavy”) strand and the 12S and 16S rRNAs for the downstream analyses, and manually removed the other regions, while visually ensuring the integrity of the alignment.

### 2.4 Phylogenetic analysis

We used beast 2.6.3 (Bouckaert et al., 2019) for simultaneous phylogeny estimation and divergence dating. As input, we used the trimmed alignment of the 12 forward (“heavy”) strand protein‐coding genes and rRNAs described above, partitioned by codon position for the protein‐coding genes and stems and loops for the rRNAs. We linked clock and tree models for all partitions, setting the clock model to relaxed log normal. Instead of setting an a priori substitution model for each partition, we used the bModelTest module (Bouckaert & Drummond, 2017) within beast2 to select the best model during the beast mcmc run. We set the tree prior to the Coalescent Bayesian Skyline model and added MRCA priors on the ages of 10 nodes based on well‐justified fossil calibrations (de Vries & Beck, 2021). Fossil calibration ages and distributions were based on de Vries and Beck (2021); specifically, we used a uniform distribution to constrain the timing of the divergences between Alouattinae and Atelinae (13.363–34.5 Ma), Callicebinae and Pitheciinae (13.032–34.5 Ma), Callitrichidae and Cebidae (13.183–34.5 mya), Cebinae and *Saimiri* (13.032–34.5 Ma), Platyrrhini and Catarrhini (33.4–56.035 Ma), Cercopithecoidea and Hominoidea (25.193–33.4 Ma) and tarsiers and anthropoids (41–66.095 Ma). Divergences between Cercopithecini and Colobini (12.47–25.2 Ma), Hominoidea and Hylobatidae (13.4–25.2 Ma), and Haplorhini and Strepsirrhini (55.935–66.1 Ma) were constrained with exponential distributions, where the minimum age was used as the offset and the mean was set to place the maximum age at the 95% quantile. The beast2 input file is available as a Supporting Information file. We ran two mcmc chains for 100 million generations each, sampling every 10,000, for a total of 20,000 trees. We assessed convergence, mixing of the chains, and ESS in tracer version 1.7.1 (Rambaut et al., 2018). We combined the chains after removing the first 25%–32% of each as burnin and constructed a maximum clade credibility tree with treeannotator version 2.6.3.

In addition to the dated tree, we constructed a maximum likelihood tree with raxml‐ng version 1.0.2 (Kozlov et al., 2019) required for use in the species delimitation program mPTP (see below). We used the same alignment and five partitions as for the beast2 analysis, assigning the GTR + G model to all partitions (Kozlov & Stamatakis, 2019), while allowing independent model parameters, and used 25 random and 25 parsimony‐based starting trees.

### 2.5 Lineage delimitation and assessment of riverine barriers

In order to determine whether speciation in Amazonian primates has been facilitated by riverine barriers, we first used multi‐rate Poisson Tree Processes (mPTP; Kapli et al., 2017) to identify major evolutionary lineages in our sample, rather than relying on existing species identifications or the identification of clades by eye. We did this because species limits within the platyrrhines are not always well‐resolved and/or are controversial (Fordham et al., 2020; Quintela et al., 2020; Zachos et al., 2013), and, in some cases, are based on the presence of river boundaries, even if it has not always been established definitively whether the river forms a species barrier. To avoid issues of circularity based on potential river‐guided species boundaries, we thus sought to delimit lineages in a way that is agnostic to the species assignment of our samples (see Everson et al., 2020 for a similar approach). Within mPTP, we implemented both the multi‐lambda and single‐lambda approaches, which provided a more and less conservative approach to lineage delimitation, respectively (Kapli et al., 2017). We used the maximum likelihood tree generated with raxml, removed outgroups with ‐‐outgroup_crop and determined minimum branch lengths with ‐‐minbr prior to the run.

For samples that had locality data available, phylogenetic relationships and results of delimitation with mPTP were projected onto sample localities with the phytools package (Revell, 2012) in r v4.1.0 (R Core Team, 2019). For any divergences between major lineages (as identified by mPTP) that are congruent with having occurred across a river boundary, we extracted all age estimates for the divergence of the relevant node from the posterior beast2 trees, to determine whether divergences across the same river occurred synchronously and coincided with published geological estimates for the timing of river formation.

## 3 RESULTS

### 3.1 Mitochondrial genome assembly

**FIGURE 2:**
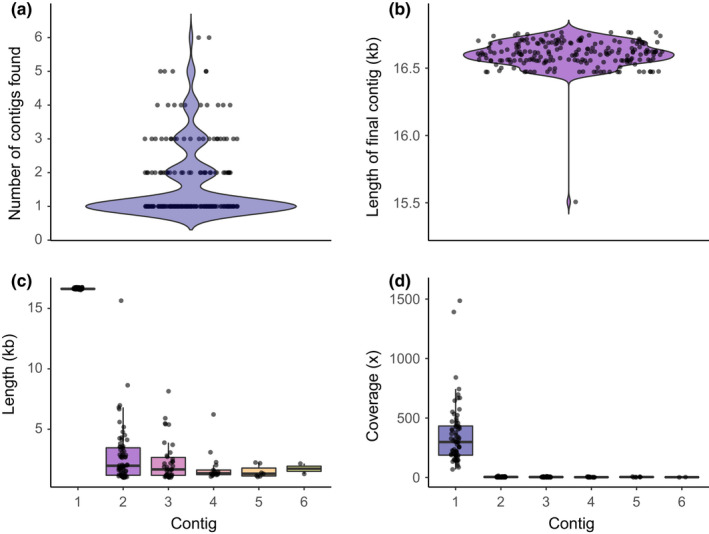
Mitochondrial genome assembly with MitoFinder. Violin plots summarize the distribution of (a) number of mitochondrial contigs found for each sample (overlaid as points) and distribution of (b) lengths of the final mitochondrial contig for all samples (overlaid as points). Boxplots describe (c) length and (d) coverage of contigs for samples in which MitoFinder identified more than one mitochondrial contig

**FIGURE 3:**
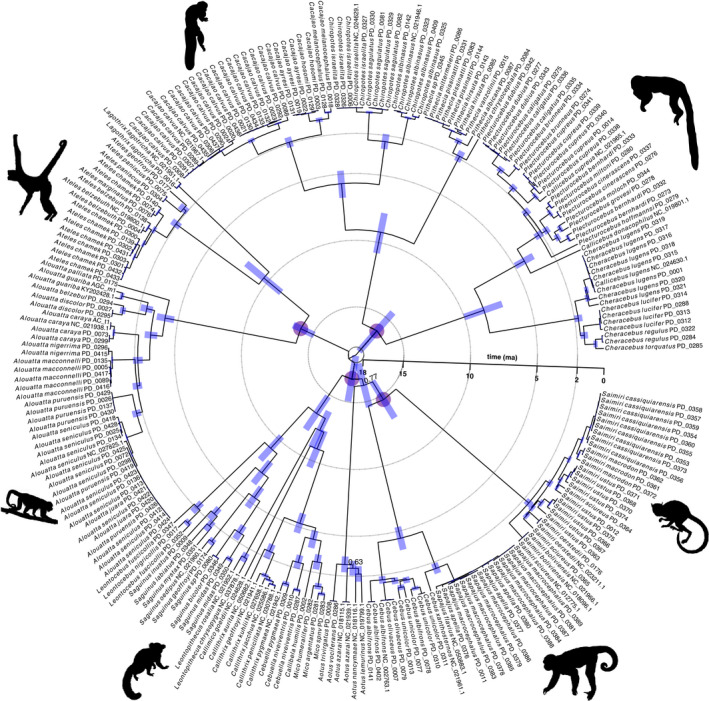
Dated mitogenomic phylogeny of platyrrhines. Blue error bars indicate 95% HPD for node ages, node numbers show posterior probability for internal nodes <0.95. Purple circles denote nodes that were calibrated with fossils. Images via PhyloPic (*Saimiri*, *Alouatta*, *Ateles*, and *Callithrix* in public domain; *Cebus* ‐ ©S. Werning), adapted from a. Cotta, cc‐by‐2.0 (Pitheciinae), adapted from B. Gratwicke, cc‐by‐2.0 (Callicebinae)

We successfully assembled complete mitochondrial genomes from Illumina short reads for all of our samples except for one (PD_0084), which was missing a small portion of the cytochrome b gene and most of the D‐loop. For the majority of our samples (135/207), only a single mitochondrial contig was assembled (Figure 2a); the final contigs across all samples had a mean length of 16,604 bp (Figure 2b), a mean coverage of 435.93x, and 15 genes were annotated for all final assemblies. In cases where multiple mitochondrial contigs were assembled (72/207), the additional contigs were always either substantially shorter (mean length of additional contigs = 2425 bp; Figure 2c) and/or had much lower coverage (mean coverage of additional contigs = 4.222×) than the first contig (mean coverage = 389.83×; Figure 2d). Only in a single case (PD_0429) was the second contig above 10 kb and resembled an almost complete mitochondrial genome. However, coverage was substantially lower for the second, shorter contig (15.32×) than for the first contig (2716.77×). While the higher‐coverage contig matched the taxon identification of the sample (*Alouatta*), the second, lower‐coverage contig matched *Saimiri*, so this probably reflects a low level of contamination. Results of the BLAST searches of the final assembled mitogenomes confirmed the taxonomic identification of the sample in all but three cases. For two samples (PD_0080, PD_0345) this is probably due to mislabelling, rather than contamination, so we retained these for the phylogenetic analysis (Figure 3), but did not consider them in downstream analyses. The third sample (PD_0305, *Ateles*) was probably contaminated, as its mitochondrial assembly was identical to another sample in a different genus (*Alouatta*), so we removed it from all analyses. Finally, two samples were found to have been collected from the same animal (PD_0306 & PD_0435), so only one sequence (PD_0435) was retained. Overall, we assembled mitochondrial genomes for samples from 17 genera (based on current nomenclature), representing all five families of platyrrhines. All final mitochondrial genomes that were newly generated and analysed in this project (*n* = 205) have been deposited in GenBank (accession numbers OM328861‐OM329065).

### 3.2 Phylogenetic analysis

Relationships within platyrrhines identified by the beast2 analysis support a basal split between Pitheciidae (sakis, uakaris, and titi monkeys) and a clade comprising the remaining families: Atelidae (howler, spider, and woolly monkeys), Cebidae (capuchin and squirrel monkeys), Callitrichidae (marmosets and tamarins), and Aotidae (owl or night monkeys). Aotidae form a clade with Cebidae in our analysis, albeit with somewhat low support (posterior = 0.77). The other phylogenetic relationships identified by the beast2 analysis are well‐supported (posterior > 0.95) for all divergences above genus level (Figure 3). The dated phylogenetic tree generated here is available in NEXUS format in the online repository containing all supporting data sets for this study (Janiak et al., 2022).

### 3.3 Lineage delimitation and assessment of riverine barriers

**FIGURE 4:**
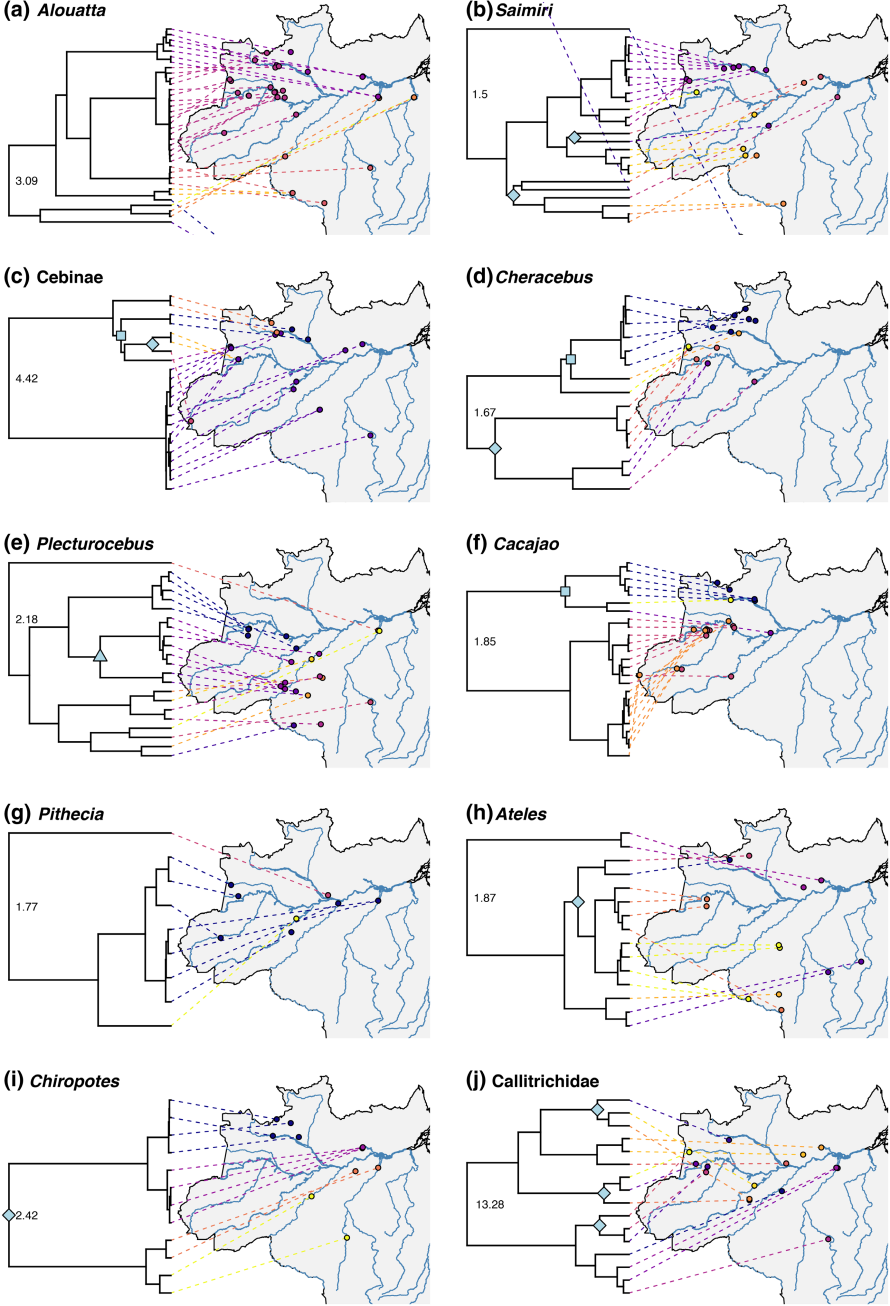
Phylogenetic relationships of platyrrhine subclades mapped onto Brazilian sampling locations. Colours indicate mPTP lineage delimitation based on the single‐rate method (see Figure S1 for multirate). Node symbols denote clades whose lineage distributions are congruent with separation by a riverine barrier, including the Amazon (diamonds), Rio Negro (squares), and Rio Madeira (triangles). Root ages for each subclade as estimated by beast2 are shown

Lineage delimitation with mPTP identified 101 distinct lineages when using a single rate of lambda (Figure 4), and 52 lineages when using the multi‐rate setting (Figure S1). We identified 13 out of a total of 64 divergences within Amazonian platyrrhines that are congruent with having occurred across a riverine barrier, meaning that members of the respective sister clades/taxa were identified as distinct lineages by mPTP and are only found on opposite sides of a river (marked with node symbols in Figure 4, Figure S1). When using the single‐rate setting, the majority of divergences that were congruent with the RBH were found for the Amazon river, including within *Saimiri, Cebus*, *Cheracebus*, *Ateles*, *Chiropotes*, and Callitrichidae (Figure 4b–d and h–j). Using the same setting, divergences that are congruent with the RBH having occurred across the Rio Negro include clades within *Cebus*, *Cheracebus*, and *Cacajao* (Figure 4c,d,f); however, in the latter two cases, only a single sample is available for the area south of the Rio Negro. For the Madeira river, the divergence between two *Plecturocebus* lineages is congruent with the RBH (Figure 4e). When using the more conservative multi‐rate setting, six of these 13 divergences are maintained out of a total 27 divergences considered. This includes the divergences within *Cebus*, *Cheracebus* and *Cacajao* across the Rio Negro (Figure S1C,D,F), within *Cheracebus* and *Chiropotes* across the Amazon (Figure S1D,I), and within *Plecturocebus* across the Madeira river (Figure S1E). However, the divergences across the Amazon within three callitrichid genera (*Saguinus*, *Cebuella*, *Leontocebus*), *Cebus*, *Saimiri*, and *Ateles* that are congruent with the RBH when using the single‐rate setting are not identified with the multi‐rate setting, as the segregating lineages are considered a single lineage in this case.

**FIGURE 5:**
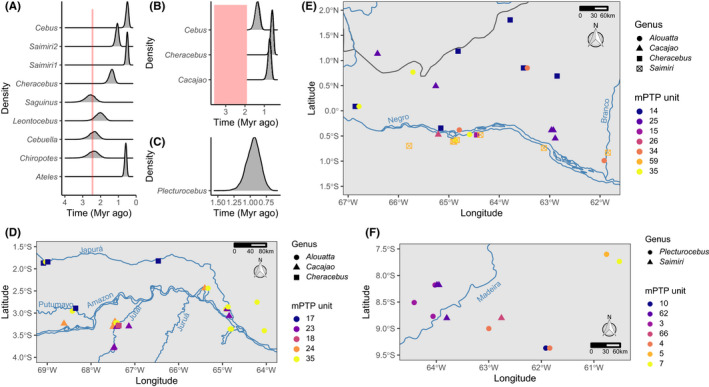
Evidence supporting the riverine barrier hypothesis is mixed for platyrrhines. Density distributions of divergence times for nodes split across the (a) Amazon, (b) Negro, and (c) Madeira rivers are only partly synchronous and many divergences postdate proposed river formation times based on geological evidence (red shading). The (d) Amazon, (e) Negro, and (f) Madeira rivers may be barriers for lineages of some genera (*Cheracebus*, *Plecturocebus*), but not for others (*Alouatta*, *Saimiri*)

The age estimates for divergences occurring across the Amazon river were partly synchronous, with 95% HPDs overlapping for the divergences identified as congruent with the RBH within *Saguinus* (2.07–3.14 Ma), *Leontocebus* (1.57–2.52 Ma), *Cebuella* (1.93–2.96 Ma), and *Chiropotes* (1.86–2.99 Ma). Divergences within *Cheracebus* (1.12–1.67 Ma), *Saimiri* (0.4–0.62 and 0.9–1.24 Ma), *Cebus* (0.36–0.65 Ma), and *Ateles* (0.48–0.7 Ma) were younger, with estimates within *Cebus*, *Saimiri*, and *Ateles* overlapping (Figure 5a). The 95% HPDs for divergences across the Rio Negro within *Cheracebus* (0.46–0.76 Ma) and *Cacajao* (0.56–0.9 Ma) overlapped, but the timing of the divergence within *Cebus* (1.13–1.61 Ma) was earlier (Figure 5b). Only a single divergence across the Madeira river was identified here, in *Plecturocebus* (0.75–1.17 Ma), so synchrony could not be assessed (Figure 5c).

For some lineages, members of the same evolutionary unit (as determined by mPTP) were found on both sides of major rivers. This included *Alouatta* across both the Amazon and the Rio Negro (Figures 5d,e), *Saimiri* across both the Rio Negro and the Madeira (Figures 5d,e), *Cacajao* across the Amazon (Figure 5d), *Sapajus* across the Amazon and Madeira (Figure 4c) and *Pithecia* across the Amazon (Figure 4g).

## 4 DISCUSSION

We assembled 205 new mitochondrial genomes for platyrrhine primates, most sampled from the Amazon region, and used them to assess support for the long‐standing riverine barrier hypothesis (RBH), which proposes that river formation was a major driver of speciation in Amazonian primates. Along the Amazon, Negro, and Madeira rivers, we found mixed evidence for the RBH, which we discuss in detail below. With the mitochondrial assemblies presented here, we have tripled the number of available mitogenomes for platyrrhines in GenBank and quadrupled the number of platyrrhine mitogenomes in RefSeq, and we provide an updated dated mitogenomic phylogeny of South American primates.

We utilized the novel mitogenomes presented here to assess support for the RBH, as originally proposed by Wallace (1852) over 150 years ago, along the Amazon, Madeira, and Negro rivers and found mixed evidence. While we identified divergences that coincide with a river barrier, only some of them occur synchronously and also overlap with the proposed dates of river formation based on geological evidence. The most compelling evidence for the RBH found here is for the Amazon river itself, within the genus *Chiropotes* (bearded saki monkeys), and also within Callitrichidae (marmosets and tamarins), which are the smallest extant platyrrhines. The Amazon river is thought to have taken its current form around 2.4–2.5 Ma (Campbell, 2010; Figueiredo et al., 2009), although it may have existed long before (11.8–11.3 Ma (Figueiredo et al., 2009); 10.0–4.5 Ma (Albert et al., 2018); 10.0–7.0 Ma (Hoorn et al., 2010)). Accepting 2.4 Ma as the minimum age of the Amazon (Campbell, 2010; Figueiredo et al., 2009), we identified four divergences in the platyrrhine tree that are congruent with the RBH and also align temporarily with this date, within the genera *Saguinus*, *Leontocebus*, *Cebuella*, and *Chiropotes*. We cautiously interpret divergences across the Amazon within these genera as being congruent with the RBH. However, we note that, despite this being the largest mitogenomic survey of the platyrrhines to date by far, some of the sample sizes are small, especially for callitrichids, and that many samples were collected some distance (~150–200 km) from the banks of the Amazon river, making it difficult to reject an alternative explanation of isolation by distance (Dambros et al., 2017). Furthermore, the relevant divergences within Callitrichidae are only supported when using the single‐rate mPTP model, not the more conservative multi‐rate model (Figure S1). That said, additional support for the Amazon as a species barrier for pygmy marmosets (*Cebuella* spp.) has recently been provided (Boubli et al., 2021; Porter et al., 2021). Notably, many divergences across the Amazon did not occur synchronously and/or postdate the known minimum time of river formation. For example, divergences within spider (*Ateles* spp.), squirrel (*Saimiri* spp.), capuchin (*Cebus* spp.) and titi (*Cheracebus* sp.) monkeys are congruent with the RBH based on sample localities, but these splits postdate even the youngest proposed date of the formation of the Amazon in its current form (2.4 Ma: Figueiredo et al., 2009) by about 1–2 million years. Additionally, we also found evidence that, despite its formidable width (~3.5 km: (Fordham et al., 2020), the Amazon does not appear to be a barrier at all for some genera, including howler monkeys (*Alouatta* spp.), bald uakaris (*Cacajao* spp.), saki monkeys (*Pithecia* spp.), and robust capuchin monkeys (*Sapajus* spp.), as members of the same evolutionary lineages occur on both sides of the river.

We identified three divergences that are congruent with the Rio Negro being a barrier, within *Cebus*, *Cheracebus*, and *Cacajao*, as suggested previously (Boubli et al., 2015). However, for both *Cacajao* and *Cheracebus* these divergences are based on a single sample present on the opposite river bank, and so need to be tested further with additional sampling. In the case of *Cebus*, the samples that form the clade south of the Rio Negro are located quite far (>500 km) from the riverbank, making it difficult to rule out alternative explanations, such as isolation by distance. Notably, we find that the Rio Negro does not appear to be a barrier for *Alouatta* or *Saimiri*, as members of the same lineage are found on both river sides. Sedimentological evidence suggests a formation date of ~3.6–1.9 Ma for the Rio Negro (Soares et al., 2017). If we accept 1.9 Ma as the minimum age for the Rio Negro, none of the divergences identified here would be temporally congruent with the RBH for this river, as the divergences within *Cebus*, *Cacajao* and *Cheracebus* are all more recent. Additionally, as noted above, the very wide (~3.5 km: Fordham et al., 2020) Amazon river does not appear to be a barrier for bald uakaris (*Cacajao* spp.) in our results, suggesting that an alternative process may explain the distribution of black uakaris along the narrower (~0.7 km: Fordham et al., 2020) Rio Negro.

Our results suggest that the Madeira river may form a barrier for titi monkeys (*Plecturocebus* spp.), as has been suggested previously (Byrne et al., 2016; Hoyos et al., 2016; Santorelli et al., 2018). However, the same river does not appear to present a barrier to squirrel monkeys (*Saimiri* spp.). Because only a single divergence congruent with the RBH was identified for the Madeira, we cannot assess synchrony here. However, the age of the Madeira river may date to the Miocene (Ruokolainen et al., 2019; Tagliacollo et al., 2015), in which case the divergence within *Plecturocebus* postdates river formation by several million years, and thus the river is unlikely to have acted as a vicariant agent. It has also been suggested that the bed of the Madeira has moved (Ruokolainen et al., 2019; Tagliacollo et al., 2015), complicating the ability to detect evidence for or against the RBH.

It is important to note that rivers can coincide with species barriers without having been vicariant agents (Naka & Pil, 2020), and that inferring evidence for or against vicariance from present‐day species ranges is based on the assumption that these ranges have not changed (Losos & Glor, 2003), which may not be the case (Graham et al., 1996). That said, we find evidence of evolutionarily distinct lineages in close geographic proximity within the same interfluve. This, along with our finding that even large Amazonian rivers do not appear to be barriers for several platyrrhine lineages, underscores the importance of other evolutionary mechanisms, beyond the RBH and allopatric speciation, for diversification within platyrrhines. While comparatively less attention has been paid to the role that mechanisms of sympatric speciation have played in shaping Amazonian primate diversity, speciation via sexual selection, ecological factors, and biotic interactions (Boughman, 2001; Dieckmann & Doebeli, 1999; Doebeli & Dieckmann, 2000; Gutiérrez et al., 2014; Maan & Seehausen, 2011; Rice & Salt, 1990) are important directions for future research. Our results are in line with several recent publications that find little or mixed evidence for the hypothesis that Amazonian rivers have been drivers of speciation, with many supporting a more nuanced, species‐specific effect of riverine barriers, rather than a global rule of rivers as vicariant agents (Kopuchian et al., 2020; Naka & Pil, 2020; Oliveira et al., 2017; Voss et al., 2019). Interestingly, at least some platyrrhines have been observed to be competent swimmers (Barnett et al., 2012; Benchimol & Venticinque, 2014; Gonzalez‐Socoloske & Snarr, 2010; Lynch Alfaro et al., 2015; Nunes, 2014), but floating islands and meandering rivers may offer another means for monkeys to cross large rivers (Ali et al., 2021; Ayres & Clutton‐Brock, 1992; Gascon et al., 2000). The distribution of Amazonian primates may be shaped by features beyond rivers, including moisture gradients (Silva et al., 2019), geological formations and soil properties (Ruokolainen et al., 2019), and vegetation patterns (Higgins et al., 2011), offering many avenues for future research directions.

Taxonomic revisions are outside of the scope of this study, and should not be based on mitochondrial (or even nuclear) data alone (Zachos et al., 2013); however, our mitogenomic phylogeny suggests that some species boundaries may need to be reassessed, as a handful of species were found to be paraphyletic, in particular within *Alouatta*. While some of these patterns may be due to incomplete lineage sorting, introgression, or hybridization, taxonomic errors are another common cause of such patterns (McKay & Zink, 2010). Mitochondrial phylogenies, like the one presented here, may be an important tool for uncovering such inconsistencies, especially in taxa for which species limits are not completely resolved, or for which ranges have been assumed to correspond to interfluvial regions or areas of endemism, which assumes a priori that rivers form dispersal barriers.

The newly assembled mitogenomes, along with their metadata, will be a valuable resource for conservation genetics and genomics, facilitating more accurate identification of sample identities and/or provenance. Novel methods to extract nuclear data and even whole genomes from low‐quality or noninvasively collected samples are available (Burrell et al., 2015; Chiou & Bergey, 2018; Fontsere et al., 2021; Orkin et al., 2021), however, the costs associated with these methods, as well as their downstream computational requirements, remain prohibitive for many researchers, especially in primate host countries. While local capacity building should be a focus for genomicists working in the Global South (de Vries et al., 2015; Hetu et al., 2019; Rodríguez et al., 2005; Şekercioğlu, 2012), these efforts will take time, and until high‐throughput methods become more accessible, mitogenomics will continue to be a pillar of conservation genomics (Pomerantz et al., 2018; Watsa et al., 2020). Importantly, the novel mitogenomes assembled here have been made publicly available on GenBank along with important metadata, including sampling locations and voucher specimens, improving their utility and value for future analyses.

## 5 CONCLUSIONS

Mitochondrial genomics remains a pillar of phylogenetics and conservation research. The 205 newly assembled mitogenomes for Amazonian primates presented here dramatically increase the number of available platyrrhine mitogenomes, and because they include known sampling locations are of additional value to future research. Using these novel mitogenomes, we find mixed support for the long‐standing riverine barrier hypothesis (RBH), supporting a more nuanced, clade‐specific effect of riverine barriers. This suggests that other evolutionary mechanisms, beyond the RBH and allopatric speciation, may also play key roles for explaining the extraordinary species‐diversity found in Amazonian primates.

## AUTHOR CONTRIBUTIONS

Mareike C. Janiak, Robin M. D. Beck, Dorien de Vries, Ian B. Goodhead, and Jean P. Boubli conceived of the study and designed the research. Mareike C. Janiak analysed the data with input from Robin M. D. Beck, Dorien de Vries, Ian B. Goodhead, and Jean P. Boubli. Lukas F. K. Kuderna, Tomàs Marquès‐Bonet, and Nicole S. Torosin generated and provided sequencing data. Nicole S. Torosin, Amanda D. Melin, Mariluce Messias, Maria N.F. da Silva, Iracilda Sampaio, Izeni P. Farias, Rogerio Rossi, Fabiano R. de Melo, João Valsecchi, Tomas Hrbek, Felipe E. Silva, and Jean P. Boubli collected and contributed samples. Mareike C. Janiak wrote the manuscript with input from all authors.

## CONFLICT OF INTEREST

Lukas F. K. Kuderna is currently an employee of Illumina Inc.

### OPEN RESEARCH BADGES

This article has earned an Open Data Badge for making publicly available the digitally‐shareable data necessary to reproduce the reported results. The data are available on figshare at https://doi.org/10.6084/m9.figshare.19606063 and https://doi.org/10.6084/m9.figshare.19610187.

## Supporting information


Figure S1
Table S1

## Data Availability

Data used or generated for this article have been made available in the European Nucleotide Archive's SRA (BioProject PRJEB49549) and NCBI's GenBank (accession numbers OM328861‐OM329065; Table S1). Files containing metadata for all samples, sequence alignments, analysis input files, and the dated phylogenetic tree have been archived at: 10.6084/m9.figshare.19606063. Scripts used to run analyses are available on GitHub (https://github.com/MareikeJaniak/Platyrrhine‐mtDNA) and have been archived at: 10.6084/m9.figshare.19610187.
