# Supplementary material for: Two hundred and five newly assembled mitogenomes provide mixed evidence for rivers as drivers of speciation for Amazonian primates
Source: Mol Ecol. 2022 Jun 20;31(14):3888–902. doi: 10.1111/mec.16554 (PMC9546496; doi:10.1111/mec.16554)
Supplement: Supplementary file 1 — Figure S1 Table S1 [file MEC-31-3888-s001.pdf]

MOLECULAR ECOLOGY

Supplemental Information for:

205 newly assembled mitogenomes provide mixed evidence for rivers as drivers of speciation for Amazonian primates

Mareike C. Janiak, Felipe E. Silva, Robin M. D. Beck, Dorien de Vries, Lukas F. K. Kuderna, Nicole S. Torosin, Amanda D. Melin, Tomàs Marquès-Bonet, Ian B. Goodhead, Mariluce Messias, Maria N. F. da Silva, Iracilda Sampaio, Izeni P. Farias, Rogerio Rossi, Fabiano R. de Melo, João Valsecchi, Tomas Hrbek, Jean P. Boubli

Table of Contents:

**Supplemental Table 1**	Page 2
**Supplemental Figure 1**	Page 14

MOLECULAR ECOLOGY

Supplemental Table 1. Samples used in this study, with voucher specimen ID or location (where available), accession number, and sample coordinates (where available). Voucher specimens are stored in the following scientific collections: Universidade Federal de Rondônia (UFRO), Instituto Nacional de Pesquisa da Amazônia (INPA), Museu Paraense Emílio Goeldi (MPEG), Instituto de Desenvolvimento Sustentável Mamirauá (IDSM), Museu Nacional do Rio de Janeiro (MN), Universidade Federal do Mato Grosso (UFMT), Museo Argentina de Ciencias Naturales Bernardino Rivadavia (MACN). Mitogenomes newly assembled for this study are labeled with "a" and references are provided for those previously published. An extended version of this table is available as a spreadsheet in the figshare repository associated with this study: DOI 10.6084/m9.figshare.19606063.

Family	Genus	Species	Sub-species	Sequencing ID	Coverage (x)	Voucher ID	Origin	Latitude	Longitude	GenBank Accession	Ref
Aotidae	Aotus	azarai	azarai	NC_018115.1			Formosa, Argentina	-26.035129	-57.989178	NC_018115.1	1
	Aotus	azarai		NC_021939.1						NC_021939.1	2
	Aotus	lemurinus		NC_019799.1						NC_019799.1	3
	Aotus	nancymaae		NC_018116.1						NC_018116.1	1
	Aotus	trivirgatus		PD_0008	767.06	INPA5719	Amazonas, Brazil	-0.8523	-63.4814	OM328869	a
	Aotus	vociferans		PD_0286	302.03	INPA7435	Amazonas, Brazil	-1.6561	-69.203	OM328942	a
Atelidae	Alouatta	belzebul		PD_0294	175.84		Pará, Brazil	-3.39	-52	OM328949	a
	Alouatta	caraya		AC_t1	32.022		Corrientes, Argentina	-27.658456	-56.074939	OM328861	a
	Alouatta	caraya		NC_021938.1						NC_021938.1	2
	Alouatta	caraya		PD_0073	197.67	UFRO576	Rondônia, Brazil	-12.3013	-63.3236	OM328891	a
	Alouatta	caraya		PD_0299	433.23	UFRO573	Rondônia, Brazil	-12.3019	-63.3218	OM328953	a
	Alouatta	discolor		PD_0027	187.13	INPA7401	Pará, Brazil	-3.4359	-55.2369	OM328889	a
	Alouatta	discolor		PD_0295	99.58		Pará, Brazil	-3.3717757	-51.974624	OM328950	a
	Alouatta	guariba	clamitans	AGC_m1	24.94	MACN52.41	Misiones, Argentina	-26.942803	-54.516458	OM328862	a
	Alouatta	guariba	clamitans	KY202428.1						KY202428.1	4
	Alouatta	juara		PD_0420	551.33		Amazonas, Brazil	-3.2159	-67.4075	OM329048	a
	Alouatta	juara		PD_0421	668.02	IDSM00085	Amazonas, Brazil	-3.2223	-67.3877	OM329049	a
	Alouatta	macconnelli		PD_0005	92.98	IDSM00080	Amazonas, Brazil	0.8523	-63.4814	OM328867	a
	Alouatta	macconnelli		PD_0089	357.71	INPA5715	Pará, Brazil	-1.4256	-56.7546	OM328907	a

MOLECULAR ECOLOGY

Family	Genus	Species	Sub-species	Sequencing ID	Coverage (x)	Voucher ID	Origin	Latitude	Longitude	GenBank Accession	Ref
	Alouatta	macconnelli		PD_0135	669.20	INPA7471	Roraima, Brazil	-0.9896	-61.8913	OM328915	a
	Alouatta	macconnelli		PD_0416	533.42	INPA7545	Pará, Brazil	-1.464	-56.7858	OM329045	a
	Alouatta	macconnelli		PD_0417	840.87	INPA7520	Amazonas, Brazil	-0.38	-64.79	OM329044	a
	Alouatta	nigerrima		PD_0296	194.59	INPA7405	Pará, Brazil	-3.3171	-55.334	OM328951	a
	Alouatta	nigerrima		PD_0415	404.16	INPA7406	Pará, Brazil	-3.3171	-55.334	OM329043	a
	Alouatta	palliata		PD_0175	12.77		Costa Rica			OM328926	a
	Alouatta	puruensis		PD_0026	1485.86	UFMT4017	Mato Grosso, Brazil	-9.9542	-56.0637	OM328888	a
	Alouatta	puruensis		PD_0137	145.62	UFRO384	Rondônia, Brazil	-8.89	-64	OM328917	a
	Alouatta	puruensis		PD_0419	412.07	INPA7499	Amazonas, Brazil	-4.9806	-62.9777	OM329047	a
	Alouatta	puruensis		PD_0426	310.99	IDSM03701	Amazonas, Brazil	-6.6937	-69.6906	OM329054	a
	Alouatta	puruensis		PD_0429	2716.77	UFRO509	Rondônia, Brazil	-13.2413	-60.3435	OM329055	a
	Alouatta	puruensis		PD_0430	697.45	UFRO577	Rondônia, Brazil	-12.3002	-63.3027	OM329058	a
	Alouatta	seniculus		NC_027825.1						NC_027825.1	5
	Alouatta	seniculus		PD_0025	497.30	INPA8497	Amazonas, Brazil	0.0878	-66.8222	OM328887	a
	Alouatta	seniculus		PD_0072	332.55	IDSM03682	Amazonas, Brazil	-2.7538	-64.21	OM328890	a
	Alouatta	seniculus		PD_0134	175.67	MN68607	Amazonas, Brazil	0.7687	-65.7076	OM328914	a
	Alouatta	seniculus		PD_0136	217.76	IDSM03369	Amazonas, Brazil	-3.3598	-64.7184	OM328916	a
	Alouatta	seniculus		PD_0298	155.06	IDSM03683	Amazonas, Brazil	-2.8606	-64.9263	OM328952	a
	Alouatta	seniculus		PD_0412	404.92	IDSM03694	Amazonas, Brazil	-2.9543	-68.4239	OM329040	a
	Alouatta	seniculus		PD_0413	236.40	INPA7430	Amazonas, Brazil	-1.8461	-69.0469	OM329041	a
	Alouatta	seniculus		PD_0414	298.07	INPA7433	Amazonas, Brazil	-1.7017	-69.1697	OM329042	a
	Alouatta	seniculus		PD_0418	223.05	INPA7530	Amazonas, Brazil	-0.5886	-64.914	OM329046	a
	Alouatta	seniculus		PD_0422	523.81	IDSM03367	Amazonas, Brazil	-2.9118	-65	OM329051	a
	Alouatta	seniculus		PD_0423	572.88	IDSM03368	Amazonas, Brazil	-2.4376	-65.3656	OM329050	a
	Alouatta	seniculus		PD_0424	257.04	IDSM03684	Amazonas, Brazil	-3.3987	-64.045	OM329053	a
	Alouatta	seniculus		PD_0425	432.56	IDSM03685	Amazonas, Brazil	-3.3598	-64.7184	OM329052	a
	Alouatta	seniculus		PD_0428	186.01		Amazonas, Brazil	-0.4715	-64.5886	OM329056	a

MOLECULAR ECOLOGY

Family	Genus	Species	Sub-species	Sequencing ID	Coverage (x)	Voucher ID	Origin	Latitude	Longitude	GenBank Accession	Ref
	Ateles	belzebuth		NC_019800.1						NC_019800.1	3
	Ateles	belzebuth		PD_0004	130.00	MN68612	Amazonas, Brazil	0.4917	-65.2717	OM328866	a
	Ateles	belzebuth		PD_0138	161.92	INPA5711	Amazonas, Brazil	0.8789	-63.4494	OM328920	a
	Ateles	chamek		PD_0074	417.91	UFMT4016	Mato Grosso, Brazil	-9.9783	-56.0814	OM328892	a
	Ateles	chamek		PD_0139	126.14	IDSM00033	Amazonas, Brazil	-3.174	-67.3895	OM328918	a
	Ateles	chamek		PD_0140	274.17	UFRO187	Rondônia, Brazil	-12.0558	-60.6726	OM328919	a
	Ateles	chamek		PD_0300	136.29	IDSM00035	Amazonas, Brazil	-3.1735	-67.3899	OM328954	a
	Ateles	chamek		PD_0301	258.93	UFRO575	Rondônia, Brazil	-12.49	-63.52	OM328956	a
	Ateles	chamek		PD_0302	24.85	UFRO479	Mato Grosso, Brazil	-13.5182	-60.4436	OM328955	a
	Ateles	chamek		PD_0303	330.87	UFRO566	Rondônia, Brazil	-12.491	-63.5241	OM328957	a
	Ateles	chamek		PD_0305 *	202.25						
	Ateles	chamek		PD_0431	319.58	IDSM00107	Amazonas, Brazil	-3.8335	-67.4276	OM329057	a
	Ateles	chamek		PD_0432	673.66	IDSM00796	Amazonas, Brazil	-7.7116	-60.5968	OM329059	a
	Ateles	chamek		PD_0433	676.88	IDSM00773	Amazonas, Brazil	-7.4607	-60.6855	OM329060	a
	Ateles	geoffroyi		PD_0177	18.92		Costa Rica			OM328927	a
	Ateles	marginatus		PD_0076	137.82		Pará, Brazil	-9	-53	OM328896	a
	Ateles	paniscus		PD_0075	327.59	INPA7477	Pará, Brazil	-1.4201	-56.7206	OM328893	a
	Ateles	paniscus		PD_0304	183.77	INPA7559	Amazonas, Brazil	-2.0713	-58.3751	OM328958	a
	Lagothrix	lagotricha		NC_021951.1						NC_021951.1	2
	Lagothrix	lagotricha		PD_0016	555.32	IDSM03702	Amazonas, Brazil	-6.9485	-69.7299	OM328878	a
Callitrichidae	Callibela	humilis		PD_0002	100.85	INPA4090	Amazonas, Brazil	-5.5	-60.4	OM328864	a
	Callimico	goeldii		NC_024628.1						NC_024628.1	6
	Callithrix	aurita		NC_050682.1						NC_050682.1	7
	Callithrix	geoffroyi		NC_021941.1						NC_021941.1	2
	Callithrix	jacchus		NC_025586.1						NC_025586.1	8
	Callithrix	kuhlii		NC_027658.1						NC_027658.1	9
	Callithrix	penicillata		NC_030788.1						NC_030788.1	10

MOLECULAR ECOLOGY

Family	Genus	Species	Sub-species	Sequencing ID	Coverage (x)	Voucher ID	Origin	Latitude	Longitude	GenBank Accession	Ref
	Callithrix	pygmaea		NC_021942.1						NC_021942.1	2
	Cebuella	niveiventris		PD_0010	227.10	IDSM00006	Amazonas, Brazil	-3.21801	-67.334296	OM328875	a
	Cebuella	niveiventris		PD_0287	646.08	IDSM00774	Amazonas, Brazil	-3.735624	-67.469317	OM328943	a
	Cebuella	pygmaea		PD_0309	321.27	INPA7253	Amazonas, Brazil	-1.842472	-69.022833	OM328960	a
	Leontocebus	fuscicollis	weddelli	PD_0347	184.99	INPA7509	Amazonas, Brazil	-6.3461	-63.4236	OM328997	a
	Leontocebus	fuscicollis		PD_0352	220.06	INPA7456	Amazonas, Brazil	-1.8653	-69.0369	OM329002	a
	Leontocebus	nigricollis		PD_0017	412.43	IDSM03699	Amazonas, Brazil	-2.9543	-68.4239	OM328879	a
	Leontopithecus	chrysopygus		NC_037878.1						NC_037878.1	11
	Leontopithecus	rosalia		NC_021952.1						NC_021952.1	2
	Mico	argentatus		PD_0281	549.69	INPA7420	Pará, Brazil	-3.3485	-55.1967	OM328938	a
	Mico	humeralifer		PD_0282	396.00	INPA7421	Pará, Brazil	-3.3129	-55.3217	OM328937	a
	Mico	spnv		PD_0283	254.07	UFMT3851	Mato Grosso, Brazil	-9.9825	-56.0725	OM328939	a
	Saguinus	bicolor		PD_0346	218.10		Amazonas, Brazil	-2.916	-59.9947	OM328996	a
	Saguinus	geoffroyi		PD_0174	68.12					OM328925	a
	Saguinus	inustus		PD_0009	121.47		Amazonas, Brazil	-0.73	-65.33	OM328871	a
	Saguinus	labiatus	rufiventer	PD_0348	709.15	INPA7502	Amazonas, Brazil	-6.2256	-63.4256	OM328998	a
	Saguinus	midas		PD_0349	316.69	INPA7491	Pará, Brazil	-1.4284	-56.7213	OM328999	a
	Saguinus	midas		PD_0350	489.82	INPA7558	Amazonas, Brazil	-2.0904	-58.4141	OM329000	a
	Saguinus	mystax		PD_0351	595.37	INPA7501	Amazonas, Brazil	-4.9845278	-62.960167	OM329001	a
	Saguinus	oedipus		NC_021960.1						NC_021960.1	2
	Saguinus	sp		PD_0080	220.11					OM328898	a
	Cebus	albifrons		NC_002763.1						NC_002763.1	12
	Cebus	albifrons		PD_0078	189.32	MPEG000832	Acre, Brazil	-8.6667	-72.7833	OM328895	a
Cebidae	Cebus	albifrons		PD_0141	145.16	MN68609	Amazonas, Brazil	0.4917	-65.2717	OM328922	a
	Cebus	albifrons		PD_0402	291.98	INPA7535	Amazonas, Brazil	-0.39	-64.78	OM329038	a
	Cebus	olivaceus		PD_0007	326.89		Amazonas, Brazil	-0.1175	-63.3542	OM328870	a
	Cebus	olivaceus		PD_0079	349.03	INPA7541	Roraima, Brazil	-1.0482	-61.8671	OM328897	a

MOLECULAR ECOLOGY

Family	Genus	Species	Sub-species	Sequencing ID	Coverage (x)	Voucher ID	Origin	Latitude	Longitude	GenBank Accession	Ref
	Cebus	unicolor		PD_0013	145.81	INPA7448	Amazonas, Brazil	-1.7092	-69.1093	OM328874	a
	Cebus	unicolor		PD_0077	298.40					OM328894	a
	Cebus	unicolor		PD_0310	136.67	IDSM03697	Amazonas, Brazil	-2.9038	-68.3571	OM328961	a
	Cebus	unicolor		PD_0311	343.32	IDSM03696	Amazonas, Brazil	-2.9038	-68.3571	OM328964	a
	Cebus	xanthosternos		NC_021961.1						NC_021961.1	2
	Saimiri	boliviensis	boliviensis	NC_018096.1			Ichilo, Bolivia	-16.383852	-64.222569	NC_018096.1	13
	Saimiri	boliviensis		NC_021966.1						NC_021966.1	2
	Saimiri	cassiquiarensis		PD_0353	430.15	MN68606	Amazonas, Brazil	-0.697	-65.7946	OM329003	a
	Saimiri	cassiquiarensis		PD_0354	280.72	INPA7458	Amazonas, Brazil	-0.4782	-64.4113	OM329007	a
	Saimiri	cassiquiarensis		PD_0355	438.46	INPA7539	Amazonas, Brazil	-1.6749	-69.281	OM329005	a
	Saimiri	cassiquiarensis		PD_0356	765.09	INPA7532	Roraima, Brazil	-0.8316	-61.8689	OM329004	a
	Saimiri	cassiquiarensis		PD_0357	698.34	INPA7523	Amazonas, Brazil	-0.6111	-64.9213	OM329006	a
	Saimiri	cassiquiarensis		PD_0358	445.20	INPA7534	Amazonas, Brazil	-0.5808	-64.9118	OM329008	a
	Saimiri	cassiquiarensis		PD_0359	762.99	INPA7459	Amazonas, Brazil	-0.6134	-64.9207	OM329009	a
	Saimiri	cassiquiarensis		PD_0360	369.65	INPA7458	Amazonas, Brazil	-1.6749	-69.281	OM329010	a
	Saimiri	cassiquiarensis		PD_0373	515.11		Amazonas, Brazil	-0.7394	-63.1144	OM329022	a
	Saimiri	macrodon		PD_0361	305.66	INPA7461	Amazonas, Brazil	-1.8434	-69.0265	OM329012	a
	Saimiri	macrodon		PD_0362	50.14	INPA7460	Amazonas, Brazil	-1.8434	-69.0275	OM329011	a
	Saimiri	macrodon		PD_0372	418.47	IDSM03693	Amazonas, Brazil	-2.9038	-68.3571	OM329020	a
	Saimiri	oerstedii		PD_0176	21.45					OM328928	a
	Saimiri	oerstedii	citrinellus	NC_023211.1			Puntarenas, Costa Rica	9.549719	-84.490432	NC_023211.1	13
	Saimiri	sciureus		NC_012775.1						NC_012775.1	14
	Saimiri	sciureus		PD_0364	85.81	INPA7495	Pará, Brazil	-1.4573056	-56.785389	OM329014	a
	Saimiri	sciureus		PD_0365	1920.66	INPA7557	Amazonas, Brazil	-2.0157	-58.3315	OM329015	a
	Saimiri	ustus		PD_0012	309.51	IDSM03690	Amazonas, Brazil	-6.0221	-61.6319	OM328873	a
	Saimiri	ustus		PD_0363	540.22	INPA7427	Pará, Brazil	-3.3544	-55.2203	OM329013	a
	Saimiri	ustus		PD_0366	280.38	UFRO358	Rondônia, Brazil	-8.8017	-62.76	OM329016	a

MOLECULAR ECOLOGY

Family	Genus	Species	Sub-species	Sequencing ID	Coverage (x)	Voucher ID	Origin	Latitude	Longitude	GenBank Accession	Ref
	Saimiri	ustus		PD_0367	305.36	UFRO567	Mato Grosso, Brazil	-13.3	-60.26	OM329017	a
	Saimiri	ustus		PD_0368	450.33	UFRO328	Rondônia, Brazil	-8.8027	-63.8	OM329018	a
	Saimiri	ustus		PD_0370	680.91	INPA7498	Amazonas, Brazil	-4.9845278	-62.960167	OM329019	a
	Saimiri	ustus		PD_0371	449.18	UFRO329	Amazonas, Brazil	-8.18	-64	OM329021	a
	Saimiri	ustus		PD_0374	371.21	UFRO428	Amazonas, Brazil	-8.18	-64	OM329023	a
	Saimiri	ustus		PD_0375	520.55	UFRO510	Mato Grosso, Brazil	-13.3	-60.26	OM329024	a
	Sapajus	apella		PD_0376	321.84	UFMT	Mato Grosso, Brazil	-10.0036	-56.0479	OM329025	a
	Sapajus	apella		PD_0377	457.76	INPA7496	Pará, Brazil	-1.4885	-56.7974	OM329026	a
	Sapajus	apella		PD_0380	146.48	INPA7554	Amazonas, Brazil	-2.0607	-58.3846	OM329029	a
	Sapajus	flavius		NC_050883.1						NC_050883.1	15
	Sapajus	macrocephalus		PD_0011	201.08	IDSM03691	Amazonas, Brazil	-2.9038	-68.3571	OM328872	a
	Sapajus	macrocephalus		PD_0378	425.68	INPA7505	Amazonas, Brazil	-5.7	-63.22	OM329027	a
	Sapajus	macrocephalus		PD_0379	181.64	IDSM03686	Amazonas, Brazil	-7.6131	-60.7922	OM329028	a
	Sapajus	macrocephalus		PD_0381	263.76		Amazonas, Brazil	-0.4782	-64.4113	OM329030	a
	Sapajus	macrocephalus		PD_0383	258.45	IDSM03698	Amazonas, Brazil	-2.8895	-68.3681	OM329031	a
	Sapajus	macrocephalus		PD_0384	460.79	INPA7533	Amazonas, Brazil	-0.6134	-64.9207	OM329032	a
	Sapajus	macrocephalus		PD_0385	366.95	INPA7514	Amazonas, Brazil	-4.9868	-62.9593	OM329033	a
	Sapajus	macrocephalus		PD_0386	172.05	IDSM03140	Amazonas, Brazil	-7.6013	-60.7593	OM329034	a
	Sapajus	macrocephalus		PD_0387	219.73	INPA7466	Amazonas, Brazil	-1.7195	-69.1155	OM329035	a
	Sapajus	macrocephalus		PD_0388	352.62	INPA7465	Amazonas, Brazil	-1.8412	-69.0318	OM329036	a
	Sapajus	macrocephalus		PD_0389	318.59	INPA7463	Amazonas, Brazil	-1.8554	-69.0282	OM329037	a
Pitheciidae	Cacajao	ayresi		PD_0019	277.48	INPA5247	Amazonas, Brazil	-0.5481	-62.9112	OM328881	a
	Cacajao	ayresi		PD_0022	239.45	INPA7553	Amazonas, Brazil	-0.3857	-62.9523	OM328884	a
	Cacajao	ayresi		PD_0131	560.04	INPA7552	Amazonas, Brazil	-0.3857	-62.9523	OM328911	a
	Cacajao	calvus		NC_021967.1						NC_021967.1	2
	Cacajao	calvus	calvus	PD_0020	478.47	IDSM00519	Amazonas, Brazil	-2.9118	-64.9346	OM328882	a
	Cacajao	calvus	rubicundus	PD_0021	565.66	IDSM00083	Amazonas, Brazil	-3.2008	-67.4226	OM328883	a

MOLECULAR ECOLOGY

Family	Genus	Species	Sub-species	Sequencing ID	Coverage (x)	Voucher ID	Origin	Latitude	Longitude	GenBank Accession	Ref
	Cacajao	calvus	novaesi	PD_0023	371.17	IDSM03671	Amazonas, Brazil	-6.8643	-70.1958	OM328885	a
	Cacajao	calvus	calvus	PD_0024	121.31	IDSM03677	Amazonas, Brazil	-3.065	-64.8444	OM328886	a
	Cacajao	calvus	ucayalii	PD_0088	1010.27	IDSM03679	Acre, Brazil	-7.4614	-73.6679	OM328906	a
	Cacajao	calvus	rubicundus	PD_0130	996.04	IDSM00082	Amazonas, Brazil	-3.2009	-67.4227	OM328909	a
	Cacajao	calvus	novaesi	PD_0132	1248.44	IDSM03669	Amazonas, Brazil	-6.8643	-70.1958	OM328912	a
	Cacajao	calvus	ucayalii	PD_0133	380.70	IDSM03678	Acre, Brazil	-7.4614	-73.6679	OM328913	a
	Cacajao	calvus		PD_0289	1645.72	IDSM03676	Amazonas, Brazil	-6.9351	-69.7379	OM328945	a
	Cacajao	calvus		PD_0290	707.53	IDSM00040	Amazonas, Brazil	-3.313	-67.3946	OM328946	a
	Cacajao	calvus		PD_0291	403.32	INPA7280	Amazonas, Brazil	-7.6059	-65.132	OM328947	a
	Cacajao	calvus		PD_0292	425.56	IDSM03665	Amazonas, Brazil	-3.2369	-68.6181	OM328948	a
	Cacajao	calvus		PD_0307	1168.39	IDSM00785	Amazonas, Brazil	-3.2981	-67.1371	OM328959	a
	Cacajao	calvus		PD_0308	12.22	IDSM00787	Amazonas, Brazil	-3.7713	-67.4502	OM328962	a
	Cacajao	calvus		PD_0434	570.58	IDSM03174	Amazonas, Brazil	-2.4376	-65.3656	OM329061	a
	Cacajao	calvus	rubicundus	PD_0435	182.86	IDSM00003	Amazonas, Brazil	-3.2995	-67.3739	OM329062	a
	Cacajao	calvus	rubicundus	PD_0306 **	36.61	IDSM00003					
	Cacajao	calvus	rubicundus	PD_0436	3283.61	IDSM00788	Amazonas, Brazil	-3.3116	-67.5476	OM329063	a
	Cacajao	calvus	rubicundus	PD_0437	1391.30	IDSM00786	Amazonas, Brazil	-3.7919	-67.4583	OM329064	a
	Cacajao	hosomi		PD_0003	701.53	MN68611	Amazonas, Brazil	0.4917	-65.2717	OM328865	a
	Cacajao	hosomi		PD_0129	708.14		Amazonas, Venezuela	1.1349	-66.417	OM328910	a
	Cacajao	melanocephalus		PD_0018	49.20	INPA5239	Amazonas, Brazil	-3.54	-61.45	OM328880	a
	Cacajao	melanocephalus		PD_0128	328.46		Amazonas, Brazil	-0.4727	-65.1706	OM328908	a
	Callicebus	cupreus		NC_021965.1						NC_021965.1	2
	Callicebus	donacophilus		NC_019801.1						NC_019801.1	3
	Callicebus	lugens		NC_024630.1						NC_024630.1	6
	Cheracebus	lucifer		PD_0288	188.33	IDSM03695	Amazonas, Brazil	-2.8912	-68.3552	OM328944	a
	Cheracebus	lucifer		PD_0312	913.28	INPA7440	Amazonas, Brazil	-1.8688	-69.0477	OM328963	a
	Cheracebus	lucifer		PD_0313	470.38	INPA7439	Amazonas, Brazil	-1.8429	-69.0275	OM328965	a

MOLECULAR ECOLOGY

Family	Genus	Species	Sub-species	Sequencing ID	Coverage (x)	Voucher ID	Origin	Latitude	Longitude	GenBank Accession	Ref
	Cheracebus	lucifer		PD_0314	742.86	IDSM02141	Amazonas, Brazil	-1.8215	-66.465	OM328966	a
	Cheracebus	lugens		PD_0001	454.40	MN68614	Amazonas, Brazil	-0.3426	-65.15	OM328863	a
	Cheracebus	lugens		PD_0315	331.29	MN68617	Amazonas, Brazil	1.8067	-63.7844	OM328967	a
	Cheracebus	lugens		PD_0316	369.45	MN68618	Amazonas, Brazil	1.1856	-64.8108	OM328969	a
	Cheracebus	lugens		PD_0317	356.82	INPA5709	Amazonas, Brazil	0.8523	-63.4814	OM328968	a
	Cheracebus	lugens		PD_0318	344.59	INPA5720	Amazonas, Brazil	0.6917	-62.8644	OM328970	a
	Cheracebus	lugens		PD_0319	1092.74	INPA8495	Amazonas, Brazil	0.0878	-66.8222	OM328972	a
	Cheracebus	lugens		PD_0320	239.98		Amazonas, Brazil	-0.4782	-64.4113	OM328971	a
	Cheracebus	lugens		PD_0321	402.30	INPA7442	Amazonas, Brazil	-1.7091	-69.1093	OM328973	a
	Cheracebus	regulus		PD_0284	237.51	IDSM00022	Amazonas, Brazil	-3.28	-67.316	OM328940	a
	Cheracebus	regulus		PD_0322	1083.39	IDSM00030	Amazonas, Brazil	-3.2815	-67.3183	OM328974	a
	Cheracebus	torquatus		PD_0285	80.75	INPA7497	Amazonas, Brazil	-4.9845	-62.9602	OM328941	a
	Chiropotes	albinasus		NC_021946.1						NC_021946.1	2
	Chiropotes	albinasus		PD_0142	425.82	INPA7415	Pará, Brazil	-3.3143	-55.3242	OM328921	a
	Chiropotes	albinasus		PD_0323	599.98	INPA4086	Amazonas, Brazil	-3.64	-57.44	OM328976	a
	Chiropotes	albinasus		PD_0325	583.40		Mato Grosso, Brazil	-9.8456	-58.2354	OM328975	a
	Chiropotes	albinasus		PD_0409	743.66	IDSM03689	Amazonas, Brazil	-5.9841	-61.5374	OM329039	a
	Chiropotes	israelita		NC_024629.1						NC_024629.1	6
	Chiropotes	israelita		PD_0006	292.64	INPA5717	Amazonas, Brazil	0.8523	-63.4814	OM328868	a
	Chiropotes	israelita		PD_0326	120.07	MN68621	Amazonas, Brazil	1.207	-64.7898	OM328979	a
	Chiropotes	israelita		PD_0327	128.51	INPA5721	Amazonas, Brazil	-0.4523	-62.724	OM328978	a
	Chiropotes	israelita		PD_0328	130.84		Amazonas, Brazil	-0.3426	-65.15	OM328977	a
	Chiropotes	sagulatus		PD_0081	204.28	INPA7483	Pará, Brazil	-1.4822	-56.8009	OM328899	a
	Chiropotes	sagulatus		PD_0082	449.25	INPA7481	Pará, Brazil	-1.4822	-56.8009	OM328900	a
	Chiropotes	sagulatus		PD_0329	1463.88	INPA7480	Pará, Brazil	-1.4885	-56.7974	OM329065	a
	Chiropotes	sagulatus		PD_0330	183.41	INPA7485	Pará, Brazil	-1.4037	-56.7595	OM328980	a
	Pithecia	albicans		PD_0087	174.60	INPA7513	Amazonas, Brazil	-4.9868	-62.9593	OM328905	a

MOLECULAR ECOLOGY

Family	Genus	Species	Sub-species	Sequencing ID	Coverage (x)	Voucher ID	Origin	Latitude	Longitude	GenBank Accession	Ref
	Pithecia	chrysocephala		PD_0084	232.42	INPA5908	Amazonas, Brazil	-2.8	-60	OM328902	a
	Pithecia	hirsuta		PD_0085	377.32	IDSM03692	Amazonas, Brazil	-2.8895	-68.3681	OM328903	a
	Pithecia	hirsuta		PD_0143	117.87	INPA7452	Amazonas, Brazil	-1.8477	-69.047	OM328923	a
	Pithecia	mittermeieri		PD_0086	687.63	INPA7425	Pará, Brazil	-3.3153	-55.3247	OM328904	a
	Pithecia	pissinatti		PD_0083	143.60	INPA4060	Amazonas, Brazil	-3.57	-59.05	OM328901	a
	Pithecia	pissinatti		PD_0144	652.87	INPA7504	Amazonas, Brazil	-6.25	-63.4203	OM328924	a
	Pithecia	pissinatti		PD_0331	128.66	INPA4089	Pará, Brazil	-3.3153	-55.3247	OM328981	a
	Pithecia	sp		PD_0345	164.72					OM328995	a
	Pithecia	vanzolinii		PD_0015	1615.77	IDSM03700	Amazonas, Brazil	-6.7799	-70.0189	OM328877	a
	Plecturocebus	bernhardi		PD_0273	135.74	MPEG045469	Rondônia, Brazil	-12.17	-63.19	OM328929	a
	Plecturocebus	bernhardi		PD_0332	359.34	UFRO413	Rondônia, Brazil	-9.3686	-61.8778	OM328982	a
	Plecturocebus	bernhardi		PD_0333	276.94	IDSM03688	Amazonas, Brazil	-5.9841	-61.5374	OM328983	a
	Plecturocebus	brunneus		PD_0274	303.02	UFRO327	Rondônia, Brazil	-9.3686	-61.8778	OM328930	a
	Plecturocebus	brunneus		PD_0334	422.26	UFRO541	Rondônia, Brazil	-9	-63	OM328985	a
	Plecturocebus	caligatus		PD_0275	1609.08	INPA6940	Amazonas, Brazil	-5.4907	-60.8246	OM328931	a
	Plecturocebus	caligatus		PD_0335	527.70	INPA7506	Amazonas, Brazil	-6.25	-63.4203	OM328984	a
	Plecturocebus	caligatus		PD_0336	463.51	INPA7507	Amazonas, Brazil	-6.25	-63.4203	OM328986	a
	Plecturocebus	cinerascens		PD_0276	150.57	UFRO195	Rondônia, Brazil	-12.0606	-60.6725	OM328932	a
	Plecturocebus	cinerascens		PD_0337	238.28	IDSM03139	Amazonas, Brazil	-7.6013	-60.7593	OM328987	a
	Plecturocebus	cupreus		PD_0014	220.51	IDSM00054	Amazonas, Brazil	-3.1395	-67.364	OM328876	a
	Plecturocebus	cupreus		PD_0338	426.29	INPA6928	Amazonas, Brazil	-3.8558	-63.8973	OM328988	a
	Plecturocebus	cupreus		PD_0339	499.32	INPA7518	Amazonas, Brazil	-4.9868	-62.9593	OM328990	a
	Plecturocebus	cupreus		PD_0340	615.48	IDSM00028	Amazonas, Brazil	-3.2045	-67.4322	OM328989	a
	Plecturocebus	cupreus		PD_0341	525.50	IDSM00102	Amazonas, Brazil	-3.7906	-67.4697	OM328991	a
	Plecturocebus	dubius		PD_0277	119.06	UFRO427	Amazonas, Brazil	-8.18	-64	OM328933	a
	Plecturocebus	dubius		PD_0342	244.82	UFRO403	Rondônia, Brazil	-8.770107	-64.010055	OM328992	a
	Plecturocebus	dubius		PD_0343	124.86	UFRO544	Amazonas, Brazil	-8.51	-64.42	OM328993	a

MOLECULAR ECOLOGY

Family	Genus	Species	Sub-species	Sequencing ID	Coverage (x)	Voucher ID	Origin	Latitude	Longitude	GenBank Accession	Ref
	Plecturocebus	grovesi		PD_0278	424.31	INPA7275	Mato Grosso, Brazil	-10.0017	-56.0423	OM328934	a
	Plecturocebus	hoffmannsi		PD_0279	133.88	INPA7408	Pará, Brazil	-3.3126	-55.3214	OM328935	a
	Plecturocebus	miltoni		PD_0280	228.24	IDSM03687	Amazonas, Brazil	-7.7364	-60.5152	OM328936	a
	Plecturocebus	moloch		PD_0344	1771.80	INPA7410	Pará, Brazil	-3.3564	-55.206	OM328994	a
Outgrp	Carlito	syrichta		NC_012774.1						NC_012774.1	14
Outgrp	Colobus	guereza		NC_006901.1						NC_006901.1	16
Outgrp	Lemur	catta		NC_004025.1						NC_004025.1	17
Outgrp	Nomascus	gabriellae		NC_018753.1						NC_018753.1	18
Outgrp	Pan	troglodytes		NC_001643.1						NC_001643.1	19
Outgrp	Papio	kindae		NC_020008.2						NC_020008.2	20

*sample removed because of contamination
**sample removed because tissue came from the same individual as PD_0345

References

a. Newly assembled for present study.

1. Babb, P. L., Fernandez-Duque, E., Baiduc, C. A., Gagneux, P., Evans, S., & Schurr, T. G. (2011). mtDNA diversity in Azara's owl monkeys (*Aotus azarai azarai*) of the Argentinean Chaco. *American Journal of Physical Anthropology*, *146*(2), 209–224.

2. Finstermeier, K., Zinner, D., Brameier, M., Meyer, M., Kreuz, E., Hofreiter, M., & Roos, C. (2013). A mitogenomic phylogeny of living primates. *PloS One*, *8*(7), e69504.

3. Hodgson, J. A., Sterner, K. N., Matthews, L. J., Burrell, A. S., Jani, R. A., Raaum, R. L., Stewart, C.-B., & Disotell, T. R. (2009). Successive radiations, not stasis, in the South American primate fauna. *Proceedings of the National Academy of Sciences of the United States of America*, *106*(14), 5534–5539.

4. Junior, E. C. S., et al. (unpublished). The complete mitocondrial genome of *Alouatta guariba clamitans*.

5. Di Fiore, A., Hodgson, J.A., Chiou, K.L. and Morales-Jimenez, A.L. (unpublished). *Alouatta seniculus* mitochondrial genome.

6. Menezes, A. N., Viana, M. C., Furtado, C., Schrago, C. G., & Seuánez, H. N. (2013). Positive selection along the evolution of primate mitogenomes. *Mitochondrion*, *13*(6), 846–851.

7. Malukiewicz, J., Cartwright, R. A., Curi, N. H. A., Dergam, J. A., Igayara, C. S., Moreira, S. B., Molina, C. V., Nicola, P. A., Noll, A., Passamani, M., Pereira, L. C. M., Pissinatti, A., Ruiz-Miranda, C. R., Silva, D. L., Stone, A. C., Zinner, D., & Roos, C. (2021). Mitogenomic phylogeny of *Callithrix* with special focus on human transferred taxa. *BMC Genomics*, *22*(1), 239.

8. Wang, W., Liu, J.-Y., Wang, H.-F., Yang, M.-Y., Liu, Q.-Y., & Ding, M.-X. (2016). The complete mitochondrial genome of white-tufted-ear marmoset, *Callithrix jacchus* (Primates: Callitrichinae). *Mitochondrial DNA. Part A, DNA Mapping, Sequencing, and Analysis*, *27*(3), 1920–1921.

9. Zhang, X., Pan, F., & Wu, Z.-W. (2016). Complete mitochondrial genome of *Callithrix kuhlii* (Primates: Callitrichinae) with phylogenetic consideration. *Mitochondrial DNA. Part A, DNA Mapping, Sequencing, and Analysis*, *27*(4), 2943–2944.

10. Malukiewicz, J., Hepp, C. M., Guschanski, K., & Stone, A. C. (2017). Phylogeny of the jacchus group of *Callithrix* marmosets based on complete mitochondrial genomes. *American Journal of Physical Anthropology*, *162*(1), 157–169.

11. de Freitas, P. D., Mendez, F. L., Chávez-Congrains, K., Galetti, P. M., Jr, Coutinho, L. L., Pissinatti, A., & Bustamante, C. D. (2018). Next-Generation Sequencing of the Complete Mitochondrial Genome of the Endangered Species Black Lion Tamarin *Leontopithecus chrysopygus* (Primates) and Mitogenomic Phylogeny Focusing on the Callitrichidae Family. *G3*, *8*(6), 1985–1991.

12. Arnason, U., Gullberg, A., Burguete, A. S., & Janke, A. (2000). Molecular estimates of primate divergences and new hypotheses for primate dispersal and the origin of modern humans. *Hereditas*, *133*(3), 217–228.

13. Chiou, K. L., Pozzi, L., Lynch Alfaro, J. W., & Di Fiore, A. (2011). Pleistocene diversification of living squirrel monkeys (*Saimiri* spp.) inferred from complete mitochondrial genome sequences. *Molecular Phylogenetics and Evolution*, *59*(3), 736–745.

14. Matsui, A., Rakotondraparany, F., Munechika, I., Hasegawa, M., & Horai, S. (2009). Molecular phylogeny and evolution of prosimians based on complete sequences of mitochondrial DNAs. *Gene*, *441*(1-2), 53–66.

15. Hao, Z., & Yi, C. (2019). The complete mitochondrial genome of *Sapajus flavius* (Blonde Capuchin). *Mitochondrial DNA. Part B, Resources*, *4*(2), 2970–2971.

16. Raaum, R. L., Sterner, K. N., Noviello, C. M., Stewart, C.-B., & Disotell, T. R. (2005). Catarrhine primate divergence dates estimated from complete mitochondrial genomes: concordance with fossil and nuclear DNA evidence. *Journal of Human Evolution*, *48*(3), 237–257.

17. Arnason, U., Adegoke, J. A., Bodin, K., Born, E. W., Esa, Y. B., Gullberg, A., Nilsson, M., Short, R. V., Xu, X., & Janke, A. (2002). Mammalian mitogenomic relationships and the root of the eutherian tree. *Proceedings of the National Academy of Sciences of the United States of America*, *99*(12), 8151–8156.

18. Chan, Y.-C., Roos, C., Inoue-Murayama, M., Inoue, E., Shih, C.-C., Pei, K. J.-C., & Vigilant, L. (2010). Mitochondrial genome sequences effectively reveal the phylogeny of *Hylobates* gibbons. *PloS One*, *5*(12), e14419.

19. Horai, S., Hayasaka, K., Kondo, R., Tsugane, K., & Takahata, N. (1995). Recent African origin of modern humans revealed by complete sequences of hominoid mitochondrial DNAs. Proceedings of the National Academy of Sciences of the United States of America, 92(2), 532–536.

20. Zinner, D., Wertheimer, J., Liedigk, R., Groeneveld, L. F., & Roos, C. (2013). Baboon phylogeny as inferred from complete mitochondrial genomes. *American Journal of Physical Anthropology*, *150*(1), 133–140.

MOLECULAR ECOLOGY

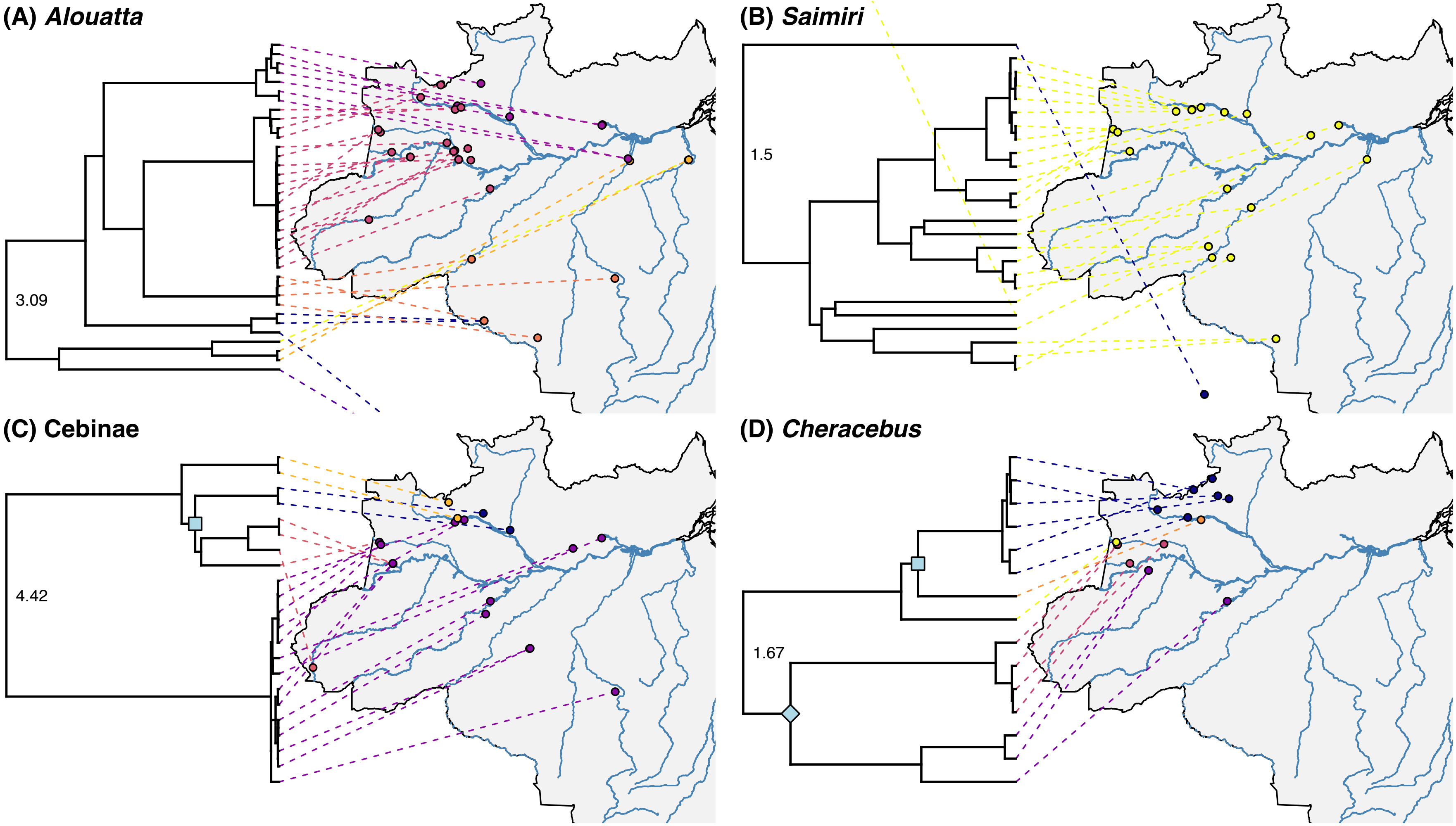

Supplemental Figure 1. Phylogenetic relationships of platyrrhine subclades mapped onto Brazilian sampling locations. Colors indicate mPTP lineage delimitation based on the multi-rate method. Node symbols denote clades whose lineage distributions are congruent with separation by a riverine barrier, including the Amazon (diamonds), Rio Negro (squares), and Rio Madeira (triangles). Root ages for each subclade as estimated by BEAST2 are shown. (Continued on next page.)

MOLECULAR ECOLOGY

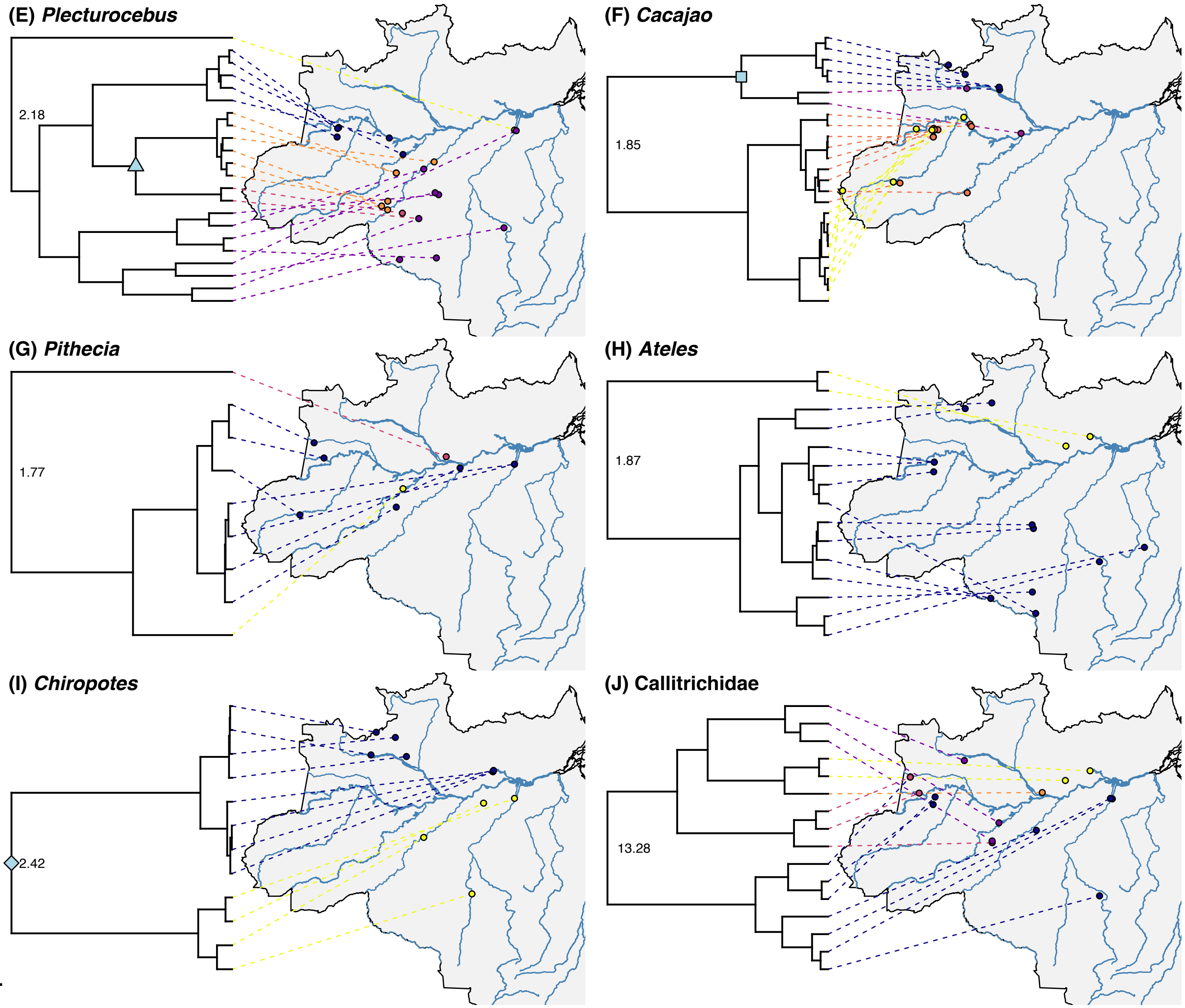

Supplemental Figure 1.
Continued from previous page.
